# Supplementary material for: Yeast Sup35 Prion Structure: Two Types, Four Parts, Many Variants
Source: Int J Mol Sci. 2019 May 29;20(11):2633. doi: 10.3390/ijms20112633 (PMC6600473; doi:10.3390/ijms20112633)
Supplement: Supplementary file 1 [file ijms-20-02633-s001.zip › Suppl exper_ijms.pdf]

Supplementary data

Involvement of the PK-protected regions in intermolecular structure. To test the involvement of individual Cores (1 to 4) in intermolecular structure, we tried to determine, which peptides remain aggregated after PK digestion. Aggregation would strongly indicate in favor of their involvement in amyloid structure, since digestion was performed in presence of strong ionic detergent sarcosyl, which dissolves almost all non-amyloid intermolecular associations (1). Aggregates are dissolved when PK digests are boiled before MS analysis. Without boiling, and assuming that the laser beam cannot break aggregates into monomers, the aggregated peptides should be absent from mass spectra. We compared spectra of boiled and unboiled digests of S-W8-R4 variant, which contains all four Cores. The amount of peptides from Regions 1 and 3 was reduced in the spectra of unboiled samples, and the difference was very sharp when the samples were loaded onto the MALDI matrix in the presence of mildly acidic volatile buffer, 50mM ammonium acetate, pH5 (Supplementary Table). However, the data for regions 2 and 4 were ambiguous. In unboiled samples the peak area was reduced for small peptides (up to 3100 Da), but increased for larger peptides. This indicates in favor of amyloid structure in Regions 1 and 3, but is uncertain about Regions 2 and 4.

Precipitation of PK digestion products with acetone, used in our procedure, showed high preference for amyloid and its fragments. Prion Sup35NMG, the same as peptides from Regions 1, 2 and 3, fully precipitated at a low (40%) concentration of acetone. For comparison, precipitation of GFP or soluble Sup35NMG required 75% acetone. Peptides from Region 4 showed intermediate properties, being present in comparable amounts in supernatant and pellet after 40% acetone. This indicates in favor of amyloid structure in Regions 1 to 3, but leaves uncertainty about Region 4.

Supplementary Table. Determination of aggregated state of individual Cores

Core 1		Core 2		Core 3		Core 4	
**Unboiled/ boiled**	**peptide**	**Unboiled/ boiled**	**peptide**	**Unboiled/ boiled**	**peptide**	**Unboiled/ boiled**	**peptide**
0,409	2-32	0,005	95-103	0,019	126-133	0,052	204-220
0,107	2-35	0,056	90-100	0,008	124-133	0,017	203-220
0,085	2-38	0,022	95-105	0,044	132-143	0,023	155-176
0,059	2-42	0,040	90-101	0,060	135-147	0,012	153-176
0,031	2-49	0,053	91-103	0,026	122-133	0,087	179-202
0,015	2-50	0,031	90-103	0,070	132-144	0,052	177-202
0,010	2-52	0,079	89-103	0,045	134-147	0,216	171-202
0,016	2-55	0,245	91-105	0,024	132-145	1,447	177-217
0,015	2-57	0,292	90-105	0,025	132-146	1,357	160-202
0,010	2-58	0,503	89-105	0,029	132-147	3,584	158-202
		0,063	95-112	0,066	132-149	1,700	177-220
		0,046	81-99	0,017	124-143	1,661	155-202
		0,087	80-99	0,052	124-147	1,571	153-202
		0,043	90-108	0,008	124-149		
		0,044	95-114	0,070	122-147		
		0,035	95-115				
		0,474	90-109				
		0,303	91-112				
		0,517	81-103				
		0,331	90-112				
		0,144	91-114				
		0,299	90-113				
		0,292	95-119				
		0,157	90-114				
		0,044	90-115				
		0,061	89-114				
		0,221	95-121				
		2,028	91-119				
		2,785	90-119				
		1,503	90-120				
		1,783	90-121				

Table shows the relation of MS peak intensities in unboiled and boiled samples, as well as peptide coordinates on the Sup35 sequence.

1. Kryndushkin DS, Alexandrov IM, Ter-Avanesyan MD, Kushnirov V V. 2003. Yeast [*PSI*+] prion aggregates are formed by small Sup35 polymers fragmented by Hsp104. J Biol Chem 278:49636–49643.
