# Supplementary material for: Yeast Sup35 Prion Structure: Two Types, Four Parts, Many Variants
Source: Int J Mol Sci. 2019 May 29;20(11):2633. doi: 10.3390/ijms20112633 (PMC6600473; doi:10.3390/ijms20112633)
Supplement: Supplementary file 1 [file ijms-20-02633-s001.zip › Fig S2_3.pdf]

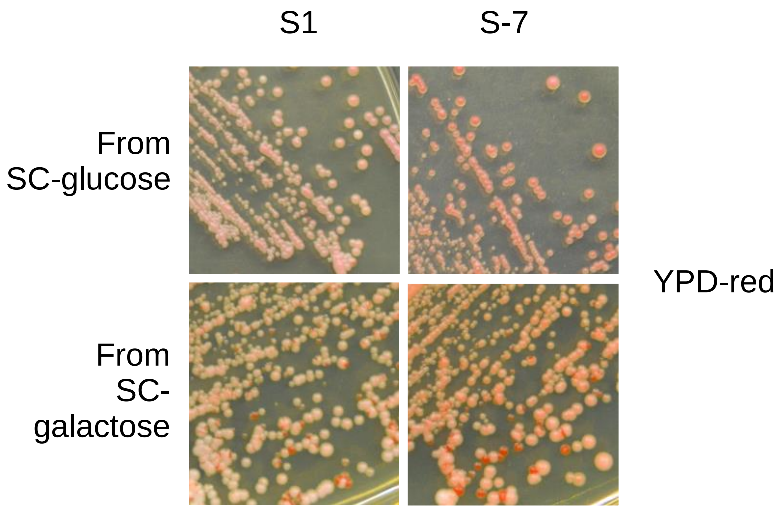

Figure S2. Yeast cells bearing S1 and S-7 [*PSI*+] variants were transformed with multicopy plasmid carrying *HSP104* and plated to SC-glucose and SC-galactose media. Figure shows transformants streaked to single cells on YPD-red medium. Red [*psi*-] colonies are observed only after SC-galactose

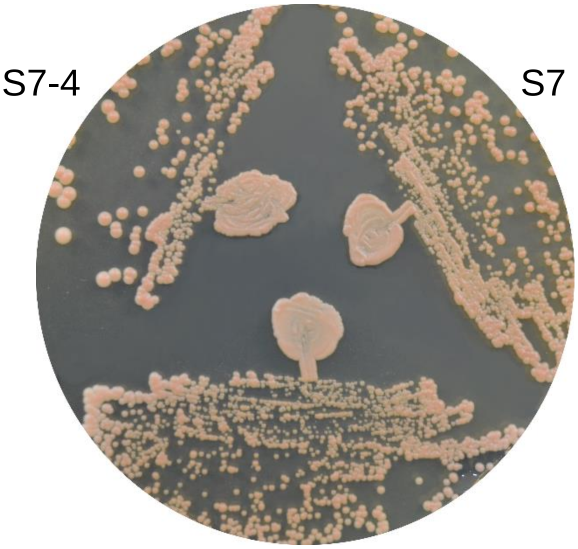

Figure S3. The S7 variant and its descendants with spontaneously altered Cores 2 and 3 are indistinguishable by colony color.
