# Supplementary material for: Yeast Sup35 Prion Structure: Two Types, Four Parts, Many Variants
Source: Int J Mol Sci. 2019 May 29;20(11):2633. doi: 10.3390/ijms20112633 (PMC6600473; doi:10.3390/ijms20112633)

Figure S1. Structures of [*PSI*+] variants. Each variant is presented by two panels. Left panel represents the general structure of Sup35NM by showing PK resistance index for the Sup35 region 2-222; right panel shows a part of MALDI-TOF mass spectrum (linear mode) containing Core 1 peptides, which gives a more detailed representation of Core 1 structure. PK resistance index was calculated as described in Materials and Methods and the text. In some variants Core 2 peptides appear to represent two separate populations, which can reflect alternative structures in this region. These populations are shown by green and orange lines, while the sum of all peptides is in blue (panels 1,8,10,24). Suffix –R14 denotes structure of prion variants after transient overproduction of Sup35N(Δ84-112)MG (panels 2,18,19,30,31). All Core 1 peptides (right panels) started from acetylated Ser2, so only the number of their C-terminal residue is given; other peptides are presented by their coordinates. Rnq1 peptides shown are 366-405 (4555.57 Da) and 360-405 (5239.24 Da).

Strong type, 74-D694

1

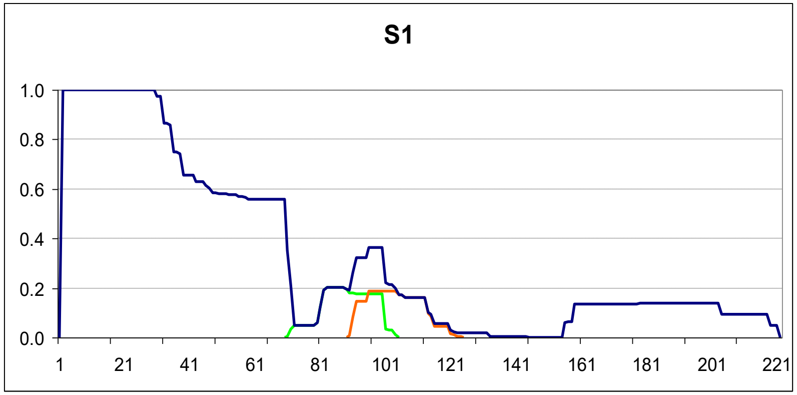

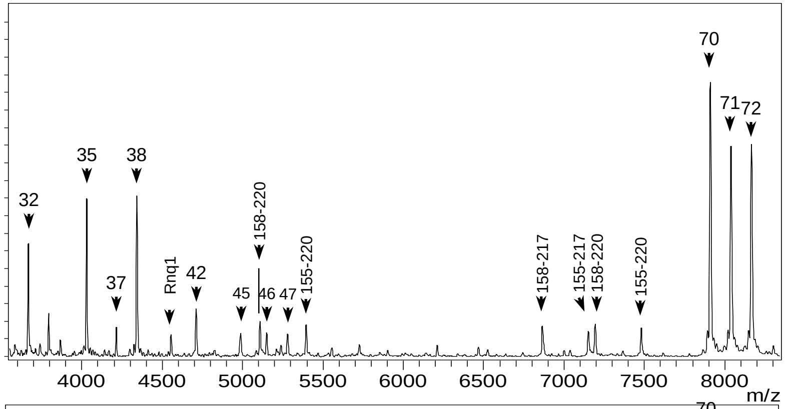

2

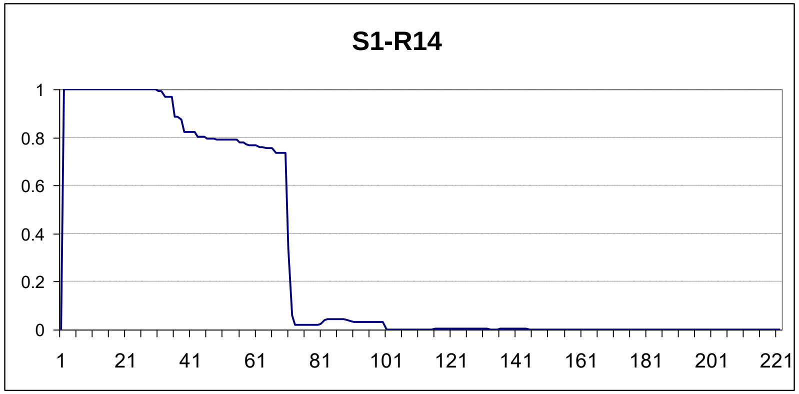

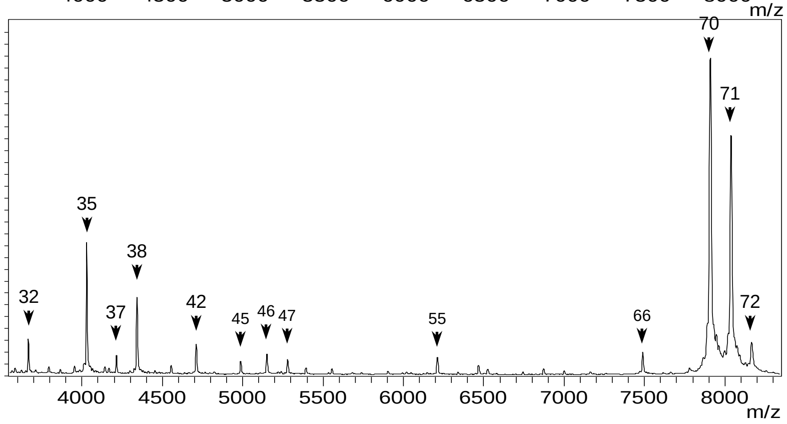

Strong type, 74-D694

3

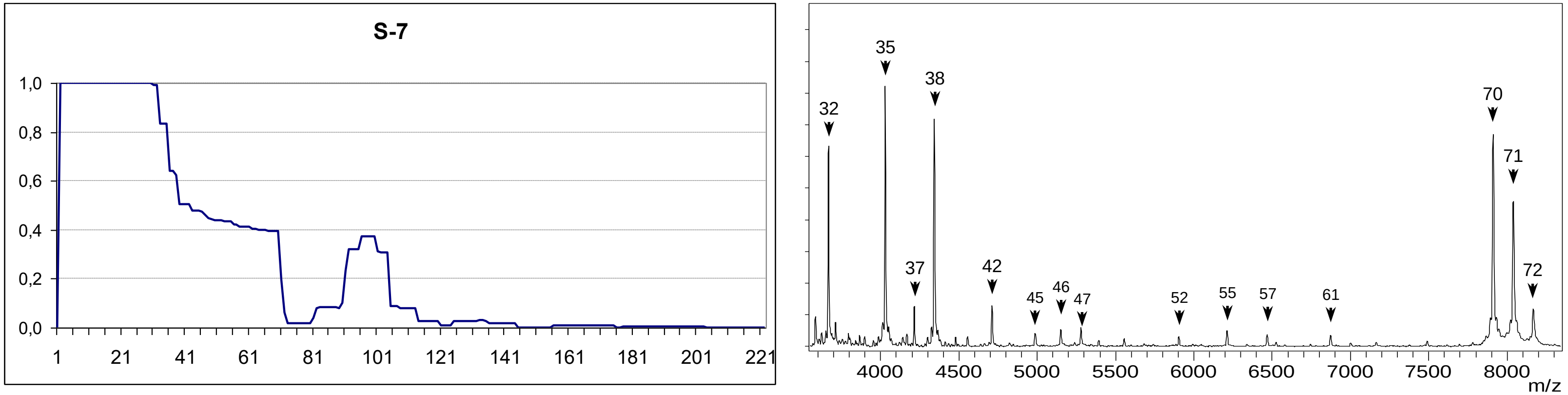

4

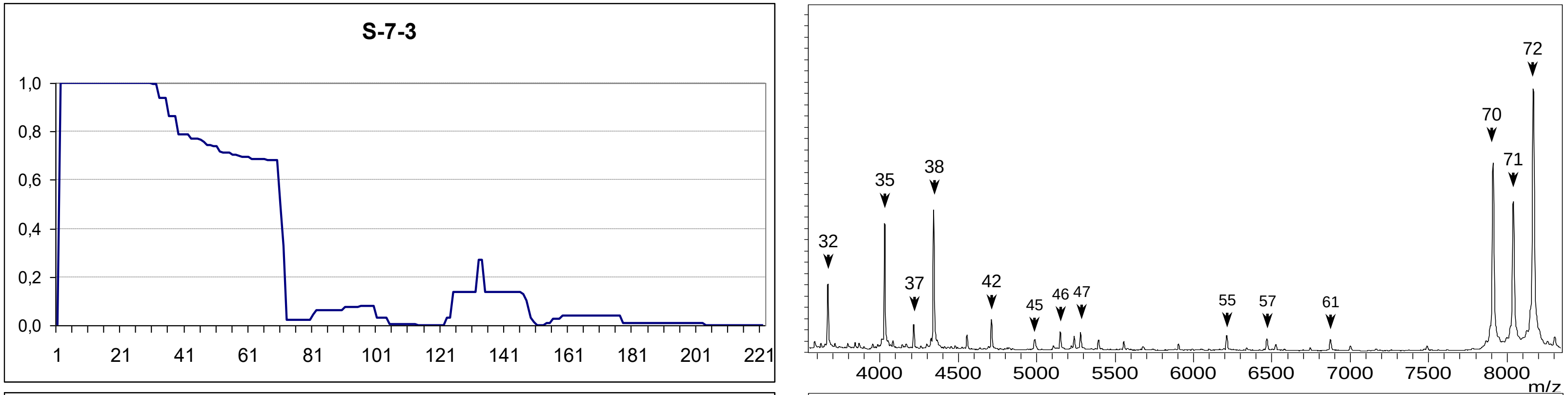

5

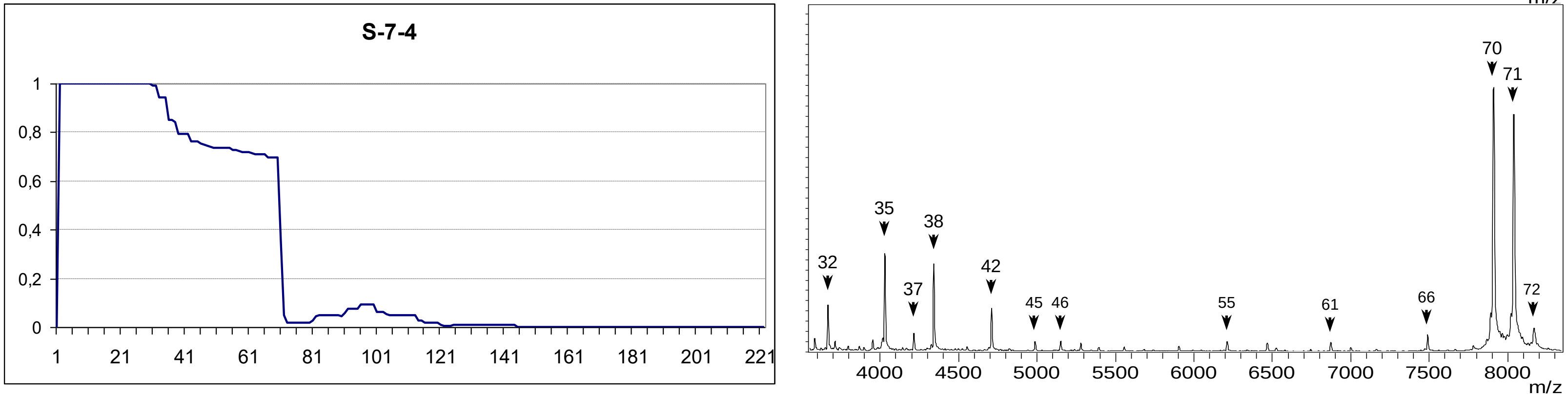

Strong type, 74-D694

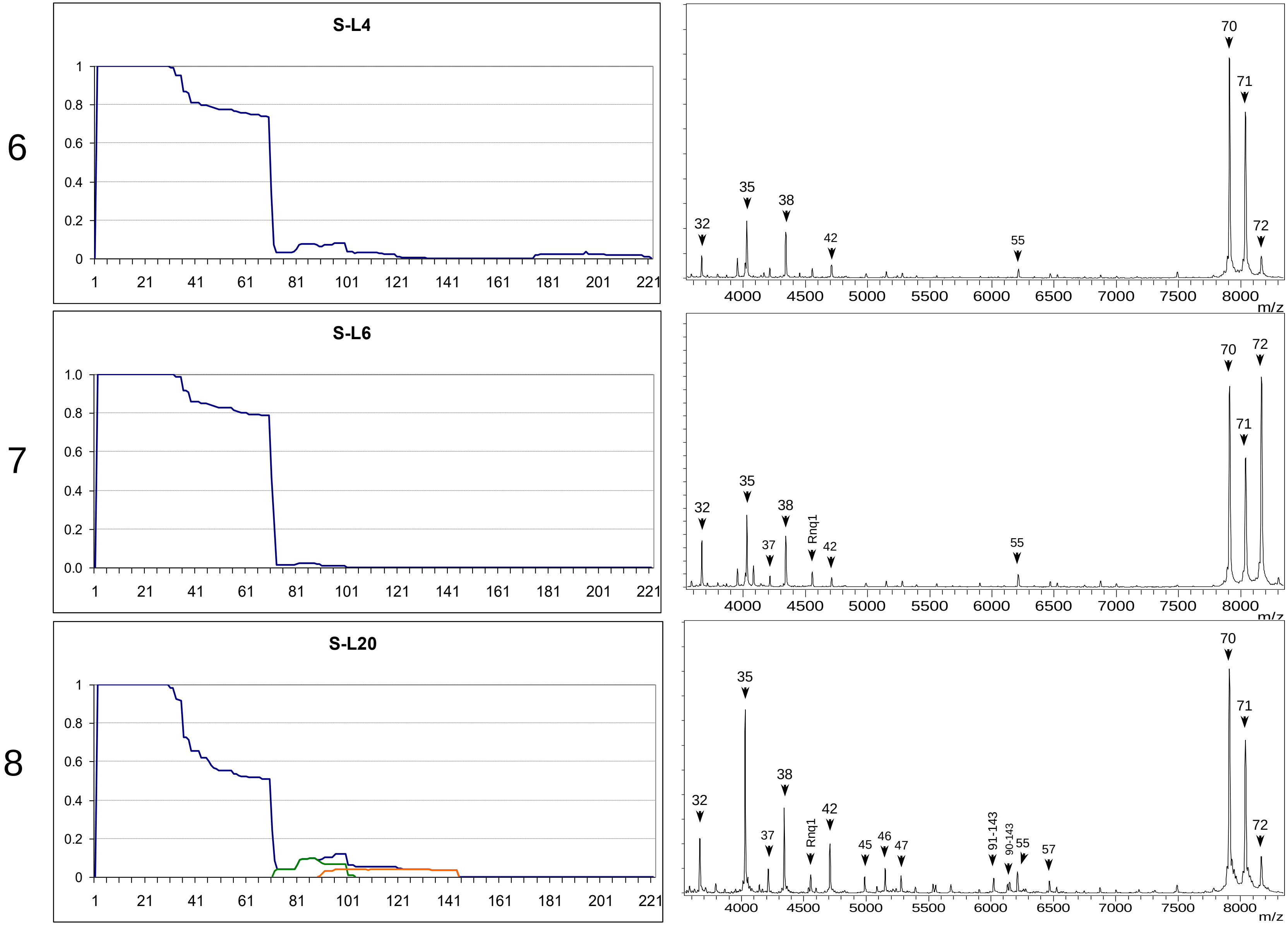

Strong type, 74-D694

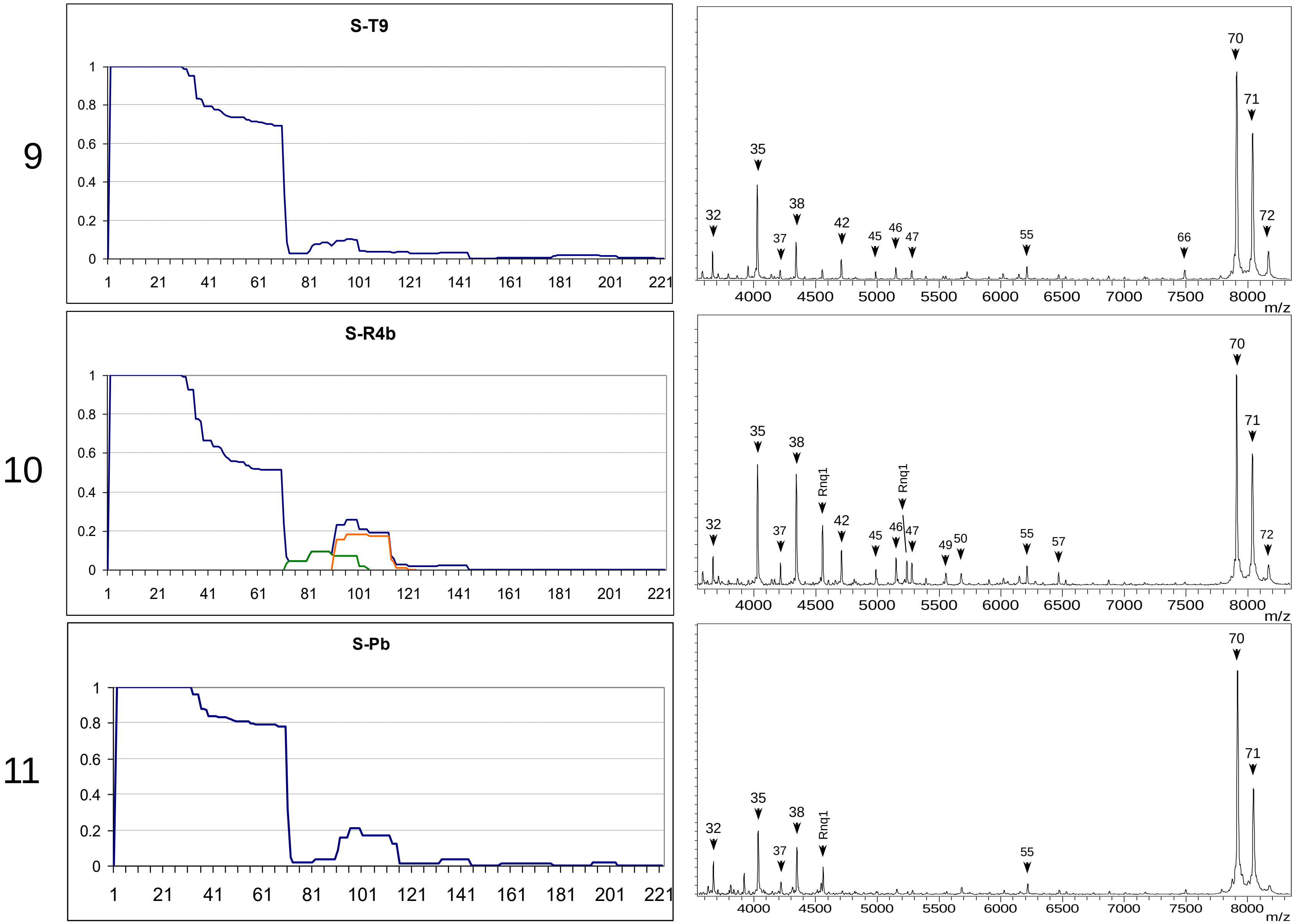

Strong type, 74-D694

12

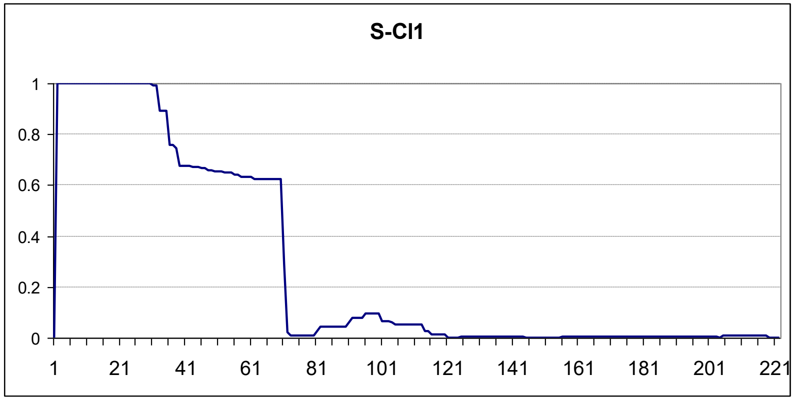

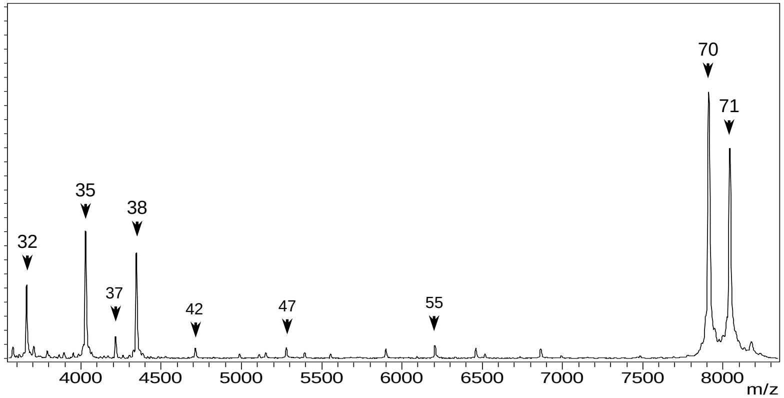

13

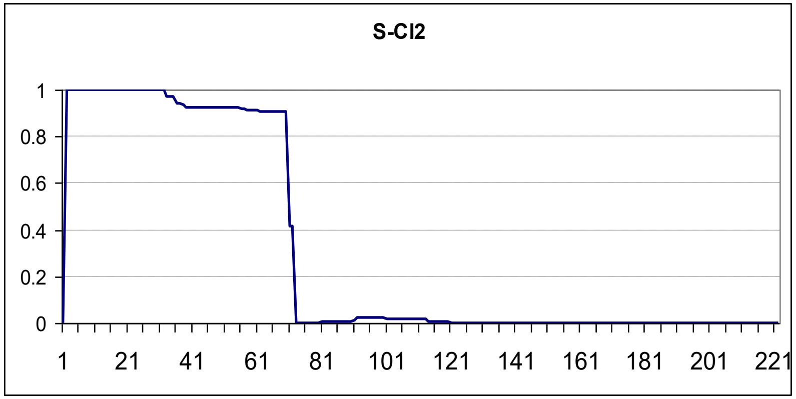

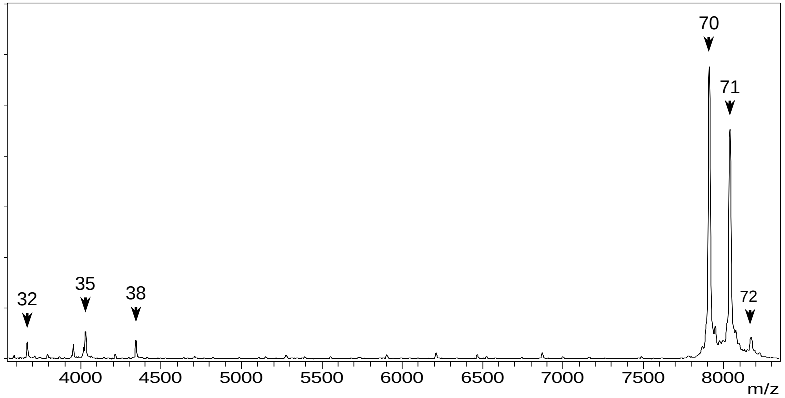

Strong type, 5V-H19

14

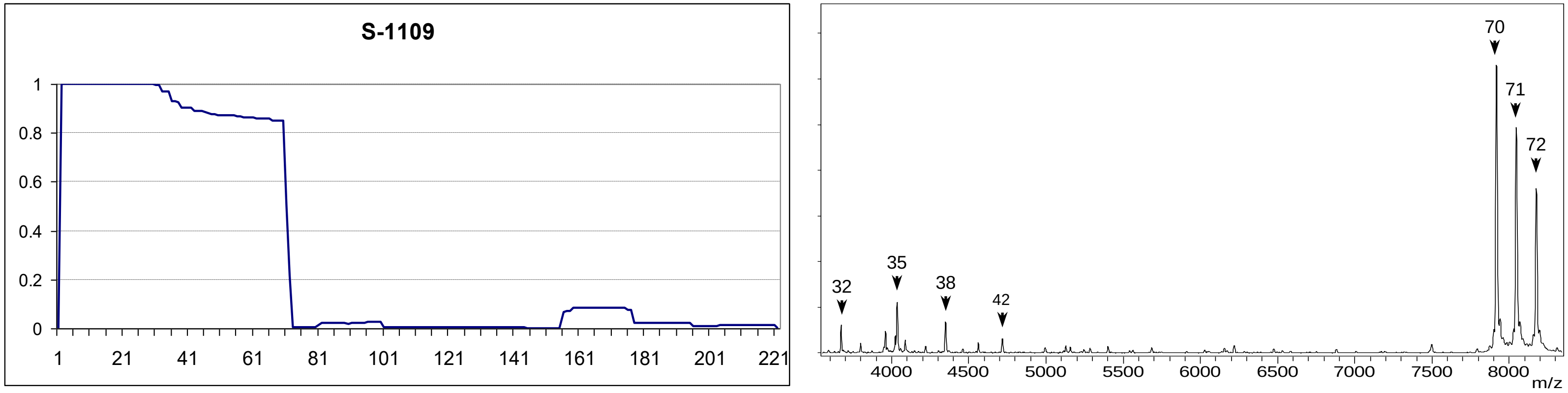

15

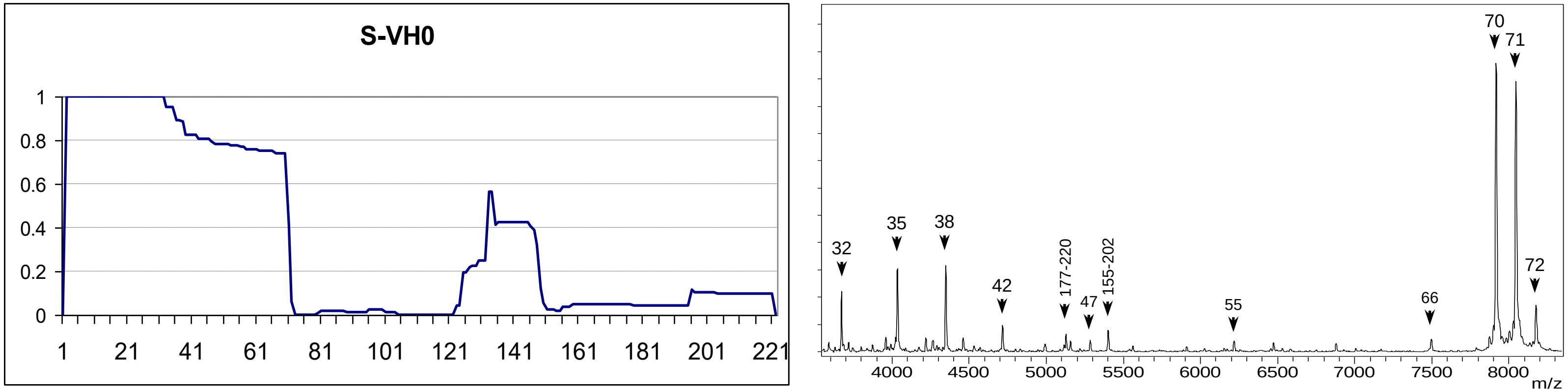

16

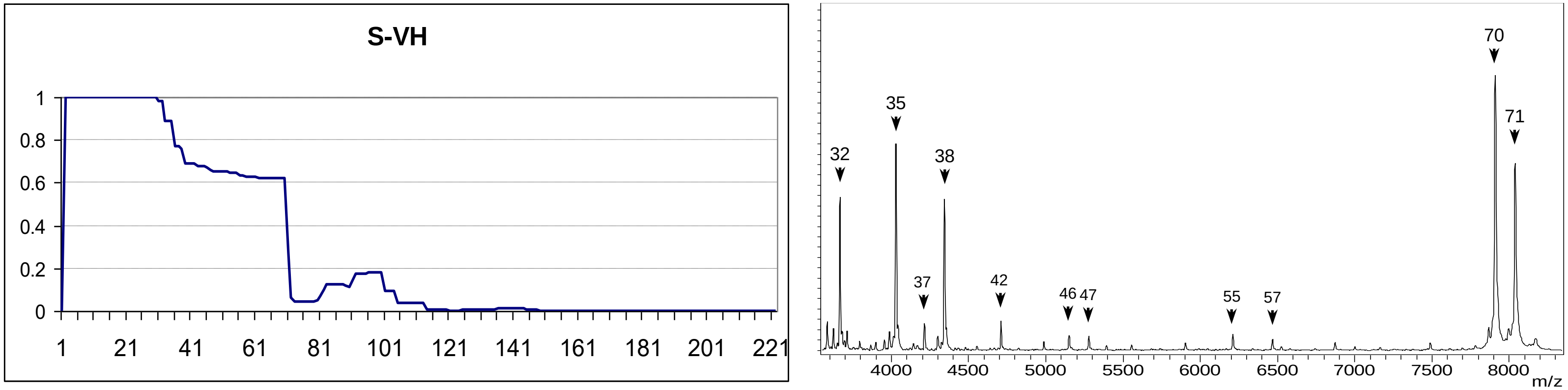

Strong type, 5V-H19

17

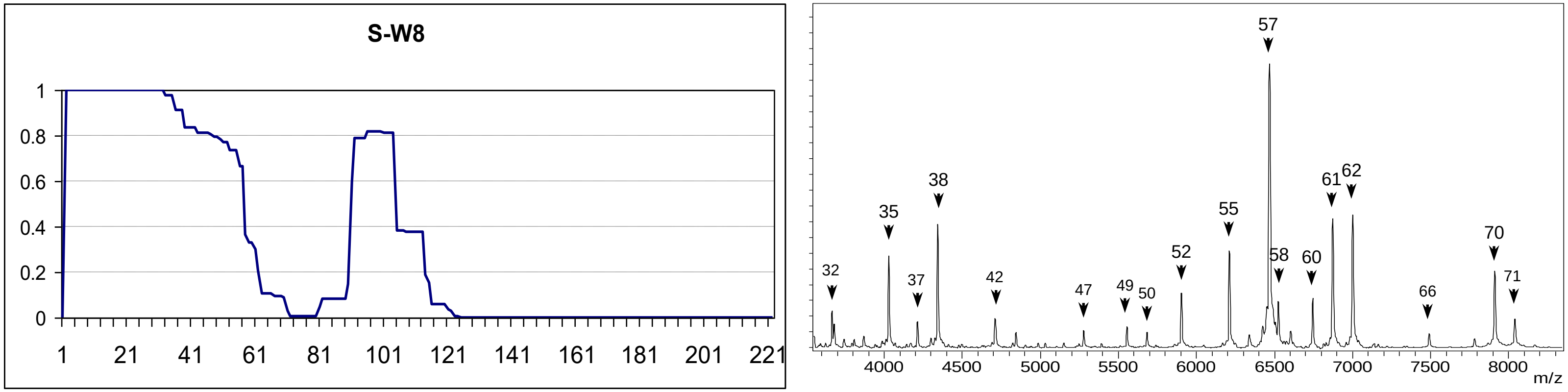

18

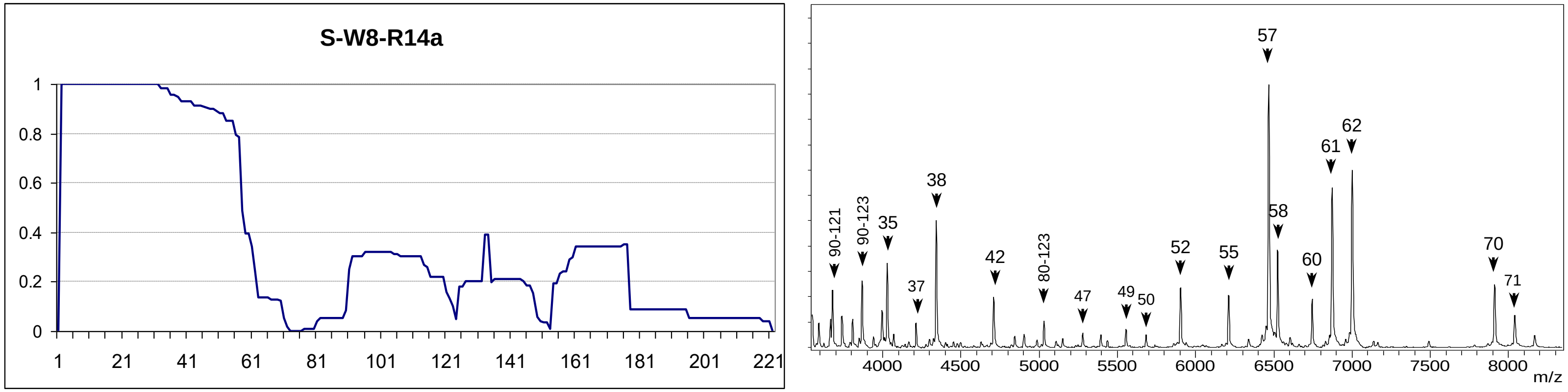

19

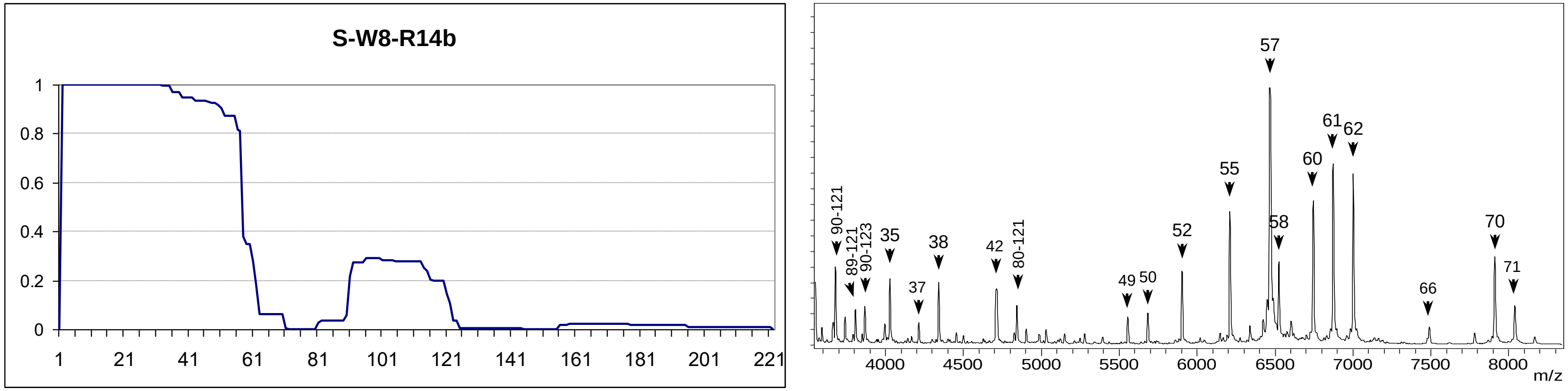

Weak type, 74-D694

20

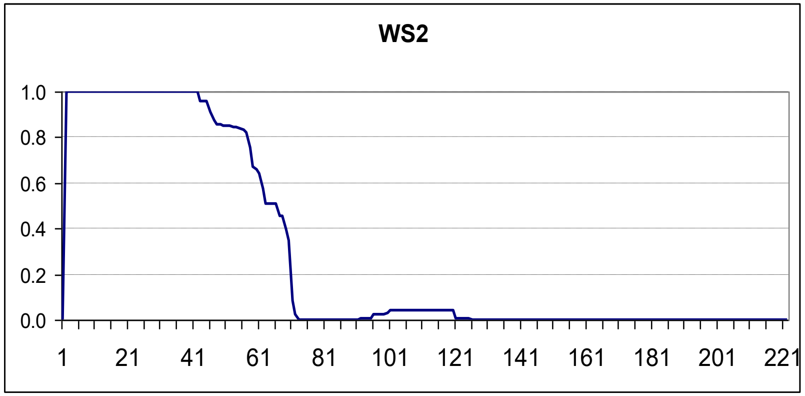

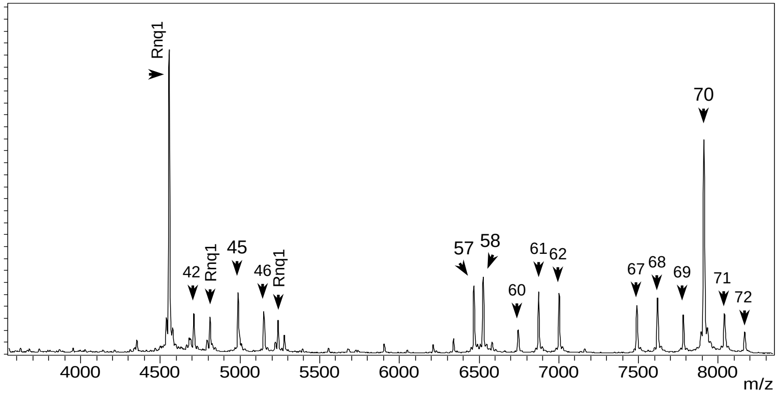

21

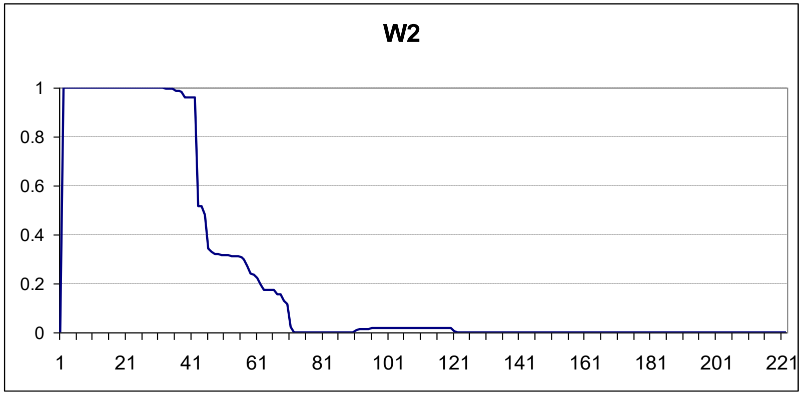

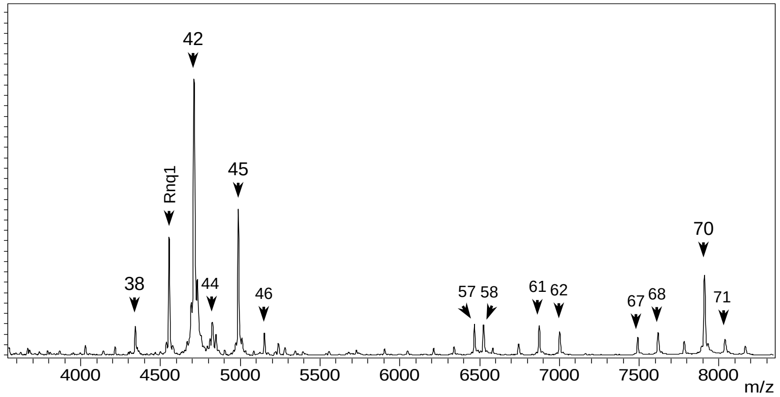

22

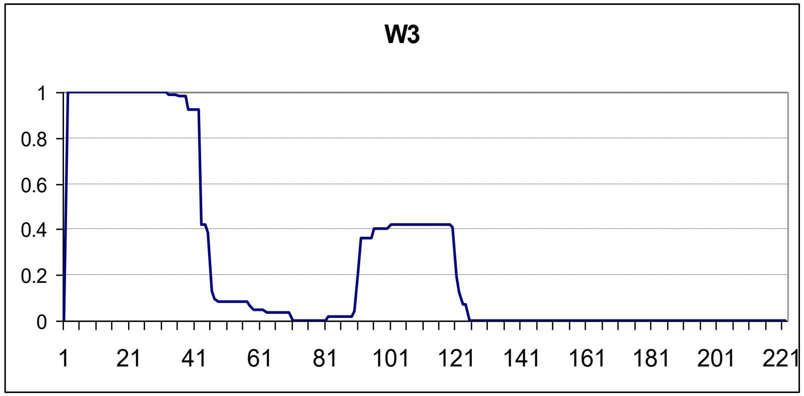

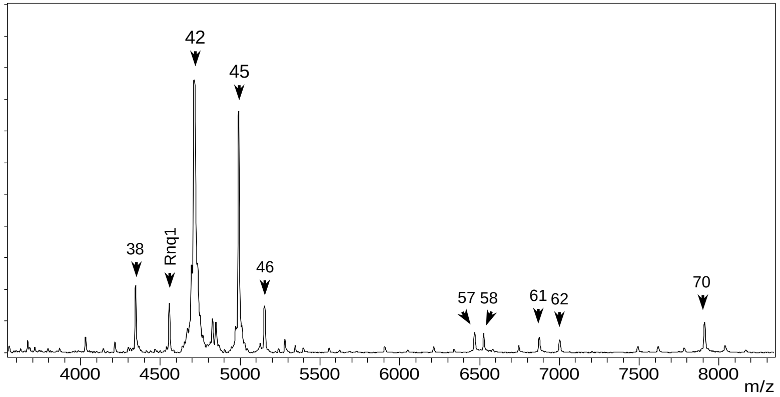

Weak type, 74-D694

23

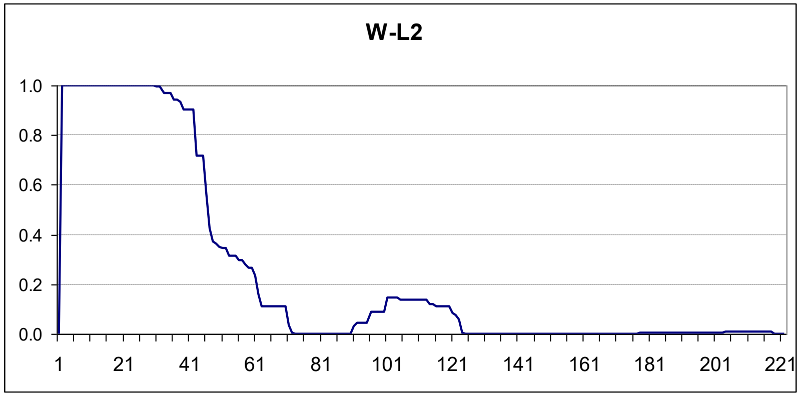

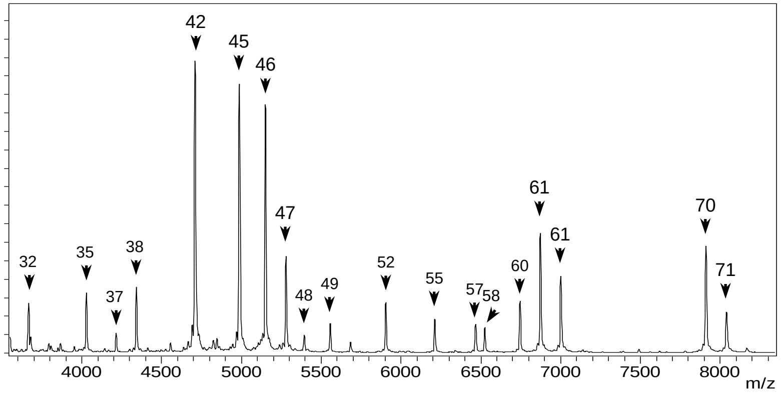

24

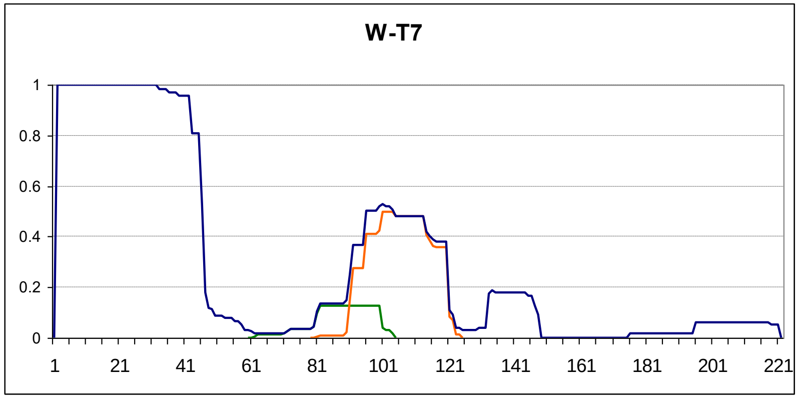

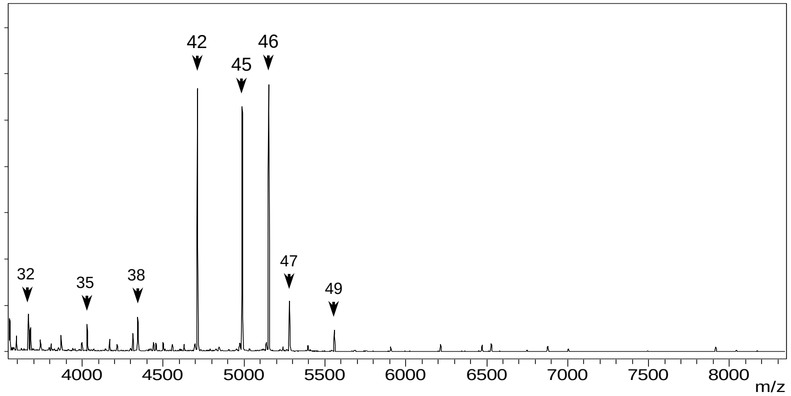

25

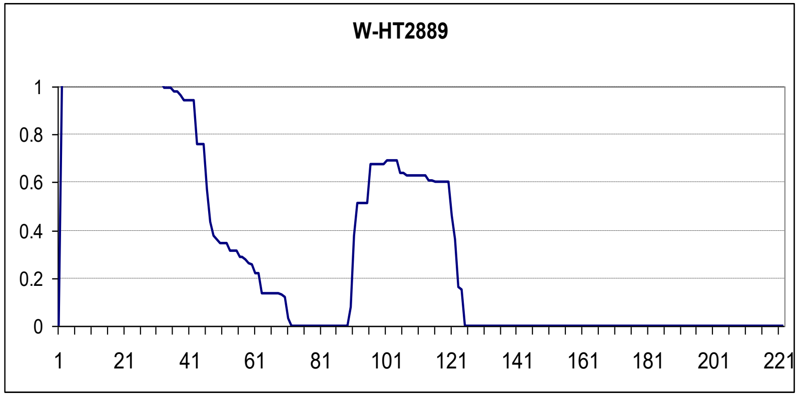

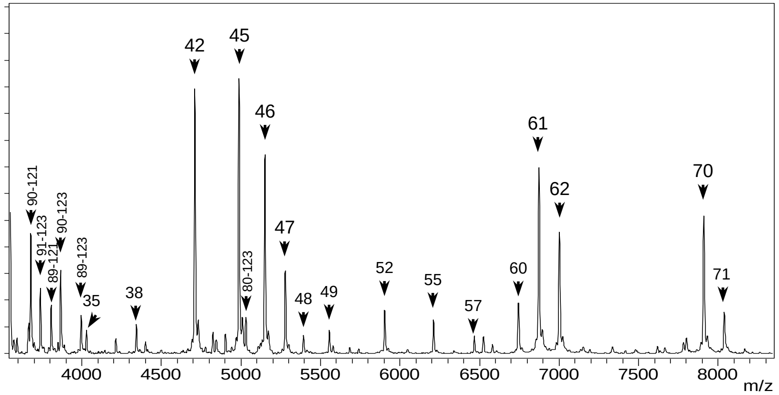

Weak type, 74-D694

26

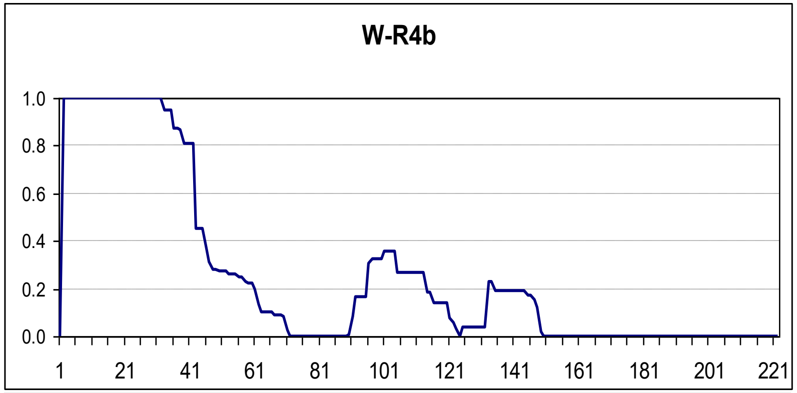

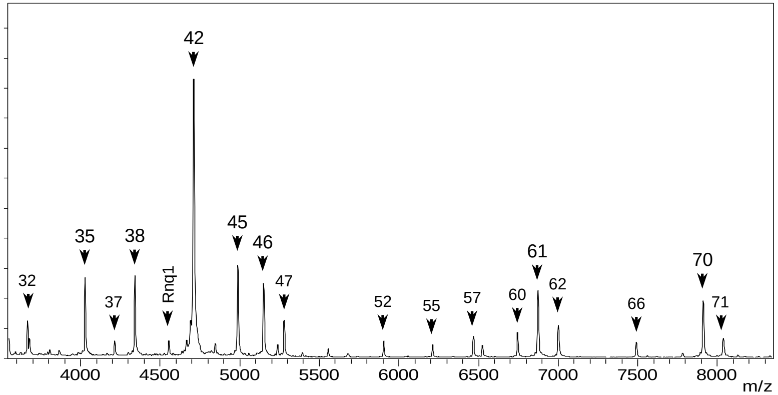

27

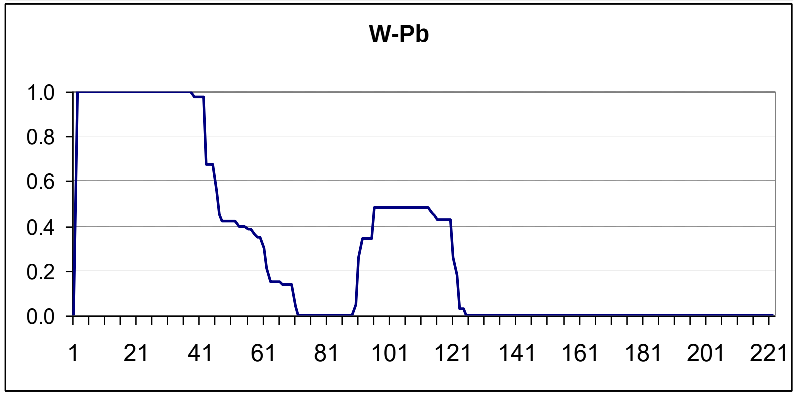

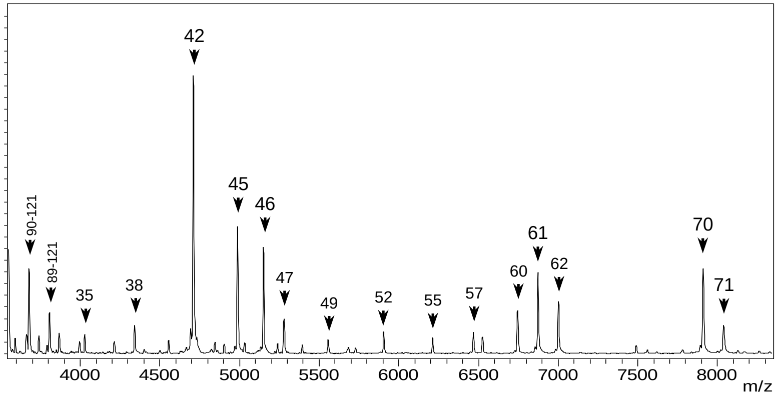

Weak type, 5V-H19

28

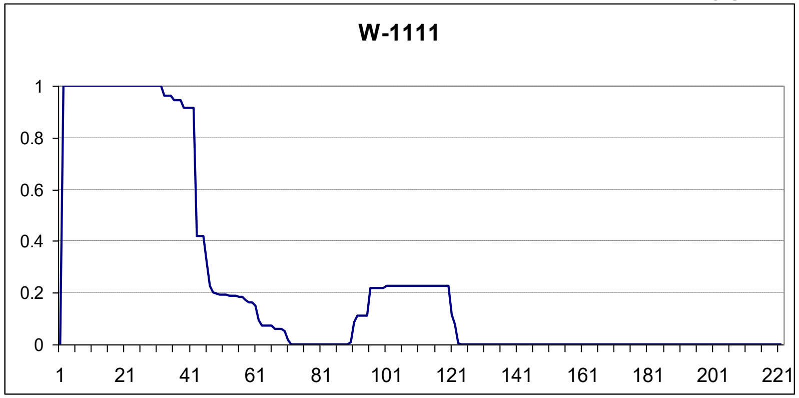

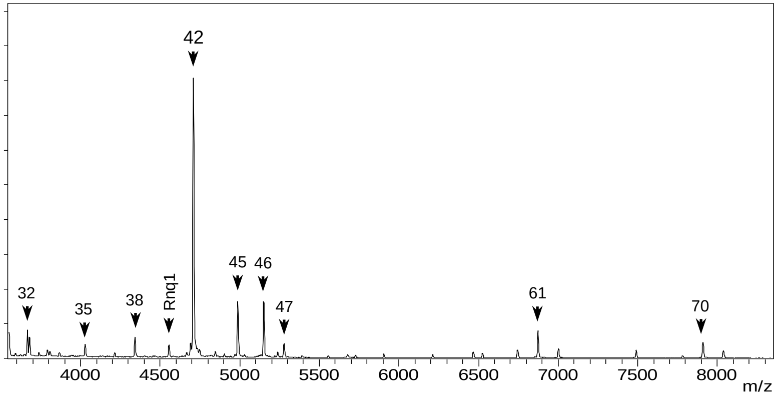

Weak type, 5V-H19

29

30

31

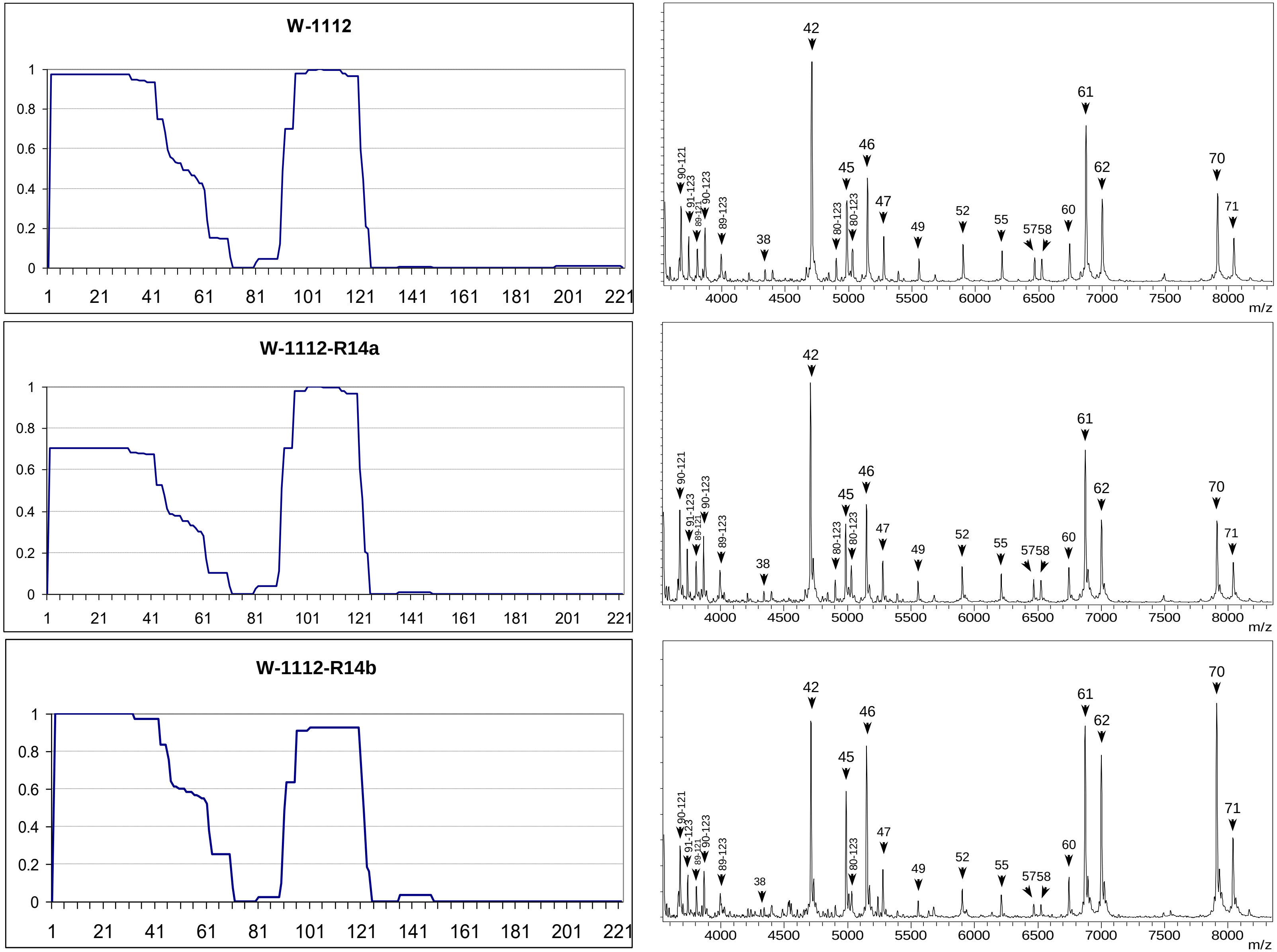

Weak type, 5V-H19

32

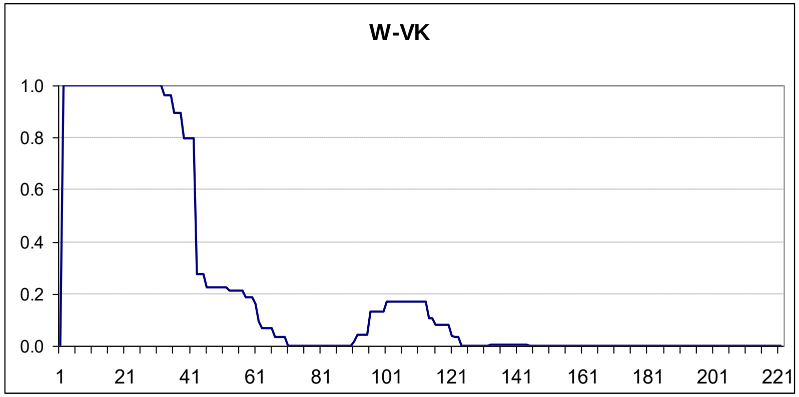

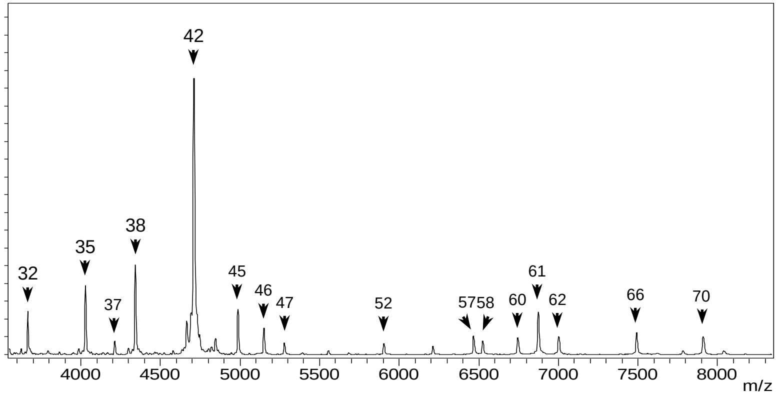

33

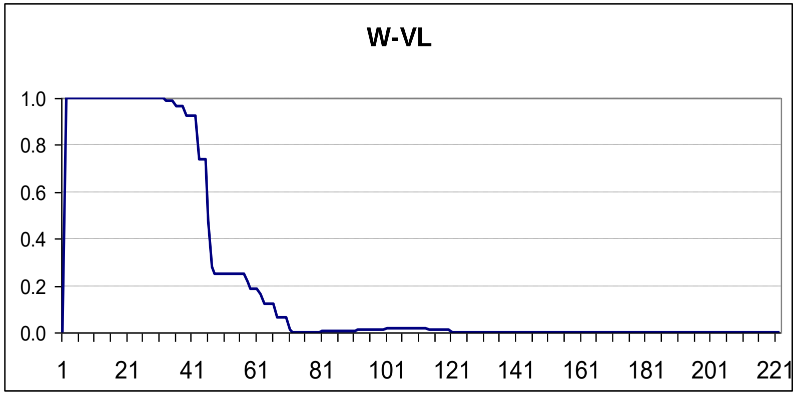

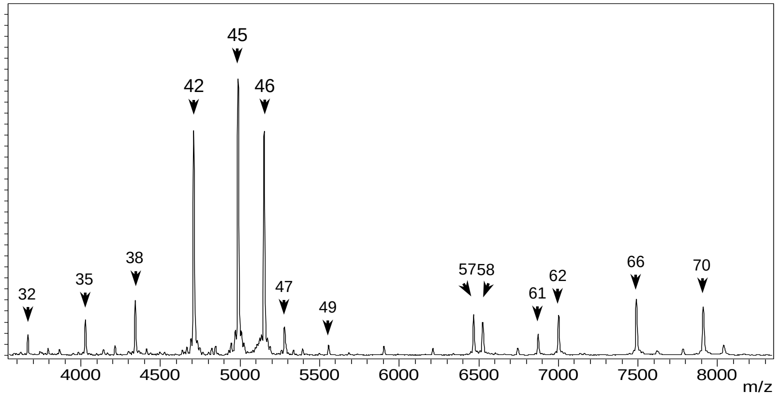

34

Amyloid of Sup35Δ(1-30)NMG from 74-D694, protein starts from residue 31

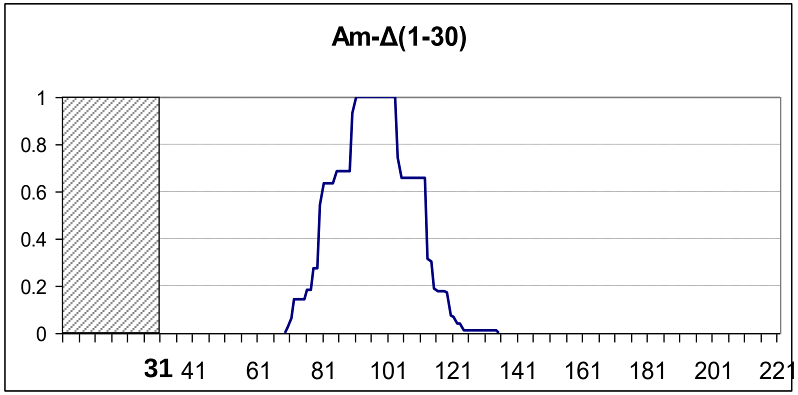

Supplement: Supplementary file 1 [file ijms-20-02633-s001.zip › FigS1_ijms.pdf]
